# Clinical assessment of the efficacy of photodynamic therapy in the treatment of oral lichen planus

**DOI:** 10.1007/s10103-012-1153-9

**Published:** 2012-07-20

**Authors:** Stefan Sobaniec, Piotr Bernaczyk, Jan Pietruski, Magdalena Cholewa, Anna Skurska, Ewa Dolińska, Ewa Duraj, Grażyna Tokajuk, Agnieszka Paniczko, Ewa Olszewska, Małgorzata Pietruska

**Affiliations:** 1 Department of Periodontal and Oral Mucosa Diseases, Medical University of Białystok, ul. Waszyngtona 13, 15-269 Białystok, Poland; 2 Department of Medical Pathomorphology, Medical University of Białystok, ul. Waszyngtona 13, 15-269 Białystok, Poland; 3 Dental Practice, ul. Waszyngtona 1/34, 15-269 Białystok, Poland; 4 Clinic of Otolaryngology, Medical University of Białystok, ul. M.C. Skłodowskiej 24a, 15-276 Białystok, Poland

**Keywords:** Oral lichen planus, Photodynamic therapy

## Abstract

The study objective was clinical assessment of the efficacy of photodynamic therapy (PDT) in the treatment of oral lichen planus (OLP). There were 23 patients aged 31–82 included in the study with oral lichen planus diagnosed clinically and histopathologically. In all patients photodynamic therapy was performed with the use of chlorin e6 (Photolon^®^), containing 20 % chlorin e6 and 10 % dimethyl sulfoxide as a photosensitizer. PDT was performed using a semiconductor laser, with power up to 300 mW and a wavelength of 660 nm. A series of illumination sessions was conducted with the use of superficial light energy density of 90 J/cm^2^. Changes of lesion size were monitored at one, two, five, and ten PDT appointments from the series of ten according to the authors' own method. The sizes of clinical OLP lesions exposed to PDT were reduced significantly (on average by 55 %). The best effects were observed for the lesions on the lining mucosa (57.6 %). The therapy was statistically significantly less effective when masticatory mucosa was affected (reduction, 30.0 %). Due to substantial efficacy and noninvasiveness, PDT can be useful in the treatment of OLP lesions.

## Introduction

Oral lichen planus (OLP) is one of the most common potentially carcinogenic chronic diseases of oral mucosa [1]. As its etiopathology cannot be established, treatment is usually symptomatic, thus showing low predictability [2–7]. In recent years, attempts have been made to introduce a new method for the treatment of lichen planus, as an alternative to standard management. This method is a photodynamic therapy (PDT), a form of photochemotherapy, using light that has a definite wavelength to activate the photosensitizer accumulated in the cells [8, 9]. One of the novel photosensitizers used in PDT is a chlorine derivative—Photolon^®^—a pigment that has been already applied in the treatment of precancerous lesions [10]. Its chemical structure corresponds to a partially reduced porphyrin moiety, whereas its molecular structure is comparable to chlorine e6, which is separated from pheophorbide during hydrolysis of 5-membered exocyclic dimethyl amine β-ketoester. Commercial Photolon^®^ contains: chlorine e6 (96.5 % purity according to high performance liquid chromatography) and polyvinylopirrolidone at 1:1 ratio.

The use of PDT has been constantly increasing due to its numerous advantages, such as selective toxicity towards inflamed or cancerous tissues, a low risk of complications, low invasiveness, and rare side effects of low intensity. At present, PDT is used for the treatment of such diseases as various types of cancers, leukoplakia, erythroleukoplakia, dysplasia, and mucosal hypertrophy. Its efficacy varies, from complete regression of lesions to the lack of response to treatment [11–15]. Besides, PDT can be an alternative to the treatment of OLP for which a universal management scheme has not yet been determined.

Taking the above into consideration, the study objective was to clinically evaluate the efficacy of photodynamic therapy in the treatment of oral lichen planus.

## Material and methods

There were 23 patients (17 women and 6 men) aged 31–82 included in the study with 48 lesions of oral lichen planus diagnosed clinically and histopathologically. Forty lesions were observed on the lining mucosa—cheeks and lips—and 8 on the masticatory—gums and tongue. Nine of the patients (three women, six men) were smokers (less than ten cigarettes per day).

To perform PDT, chlorin-e6-Photolon^®^ (Haemato Poland) was used as a photosensitizer. The gel containing 20 % chlorine e6 and 10 % dimethyl sulfoxide was applied directly onto the lesion and the surrounding healthy mucosa 1 h before illumination, using an occlusive dressing according to the authors' own method. A sheet of nonwoven fabric, exceeding the lesion in size by 5–10 mm, was carefully covered with a 1- to 2-mm layer of the photosensitizer. Next, saliva was removed from the vicinity of the lesion, and the prepared fabric was placed on dried mucosa. Another layer was composed of a polyethylene sheet of the same size as the nonwoven fabric. Finally, the dressing was additionally stabilized with a few layers of sterile gauze.

PDT was performed using a semiconductor laser Haemato LS PDT 660 (Haemato Poland). Application of 660 nm wavelength was transmitted to the lesion via an optical fiber equipped with a diffuser tip. The laser power from the end of the optical fiber did not exceed 300 mW. A series of illuminations was performed using superficial light energy density of 90 J/cm^2^. The appointments were scheduled at 2-week intervals, but no longer than for ten sessions. As there was no early response to the treatment, all patients had to undergo ten PDT sessions.

The treatment efficacy was assessed macroscopically at one, two, five, and ten appointments. Changes in the lesion sizes were monitored at the respective PDT sessions according to the authors' own method using measurement (in millimeters) with a calibrated periodontal probe PCPUNC15 (Hu-Friedy, IL, USA). To ensure repeatability of measurements, the authors' own model was used, which was designed for the need of the study to assess the lesions according to their size:

Group 0 Lack of evident lesions
Group 1 A lesion smaller than 3 cm^2^
Group 2 A lesion from 3 to <6 cm^2^
Group 3 A lesion from 6 to <10 cm^2^
Group 4 A lesion from 10 to <15 cm^2^
Group 5 A lesion >15 cm^2^

In order to analyze age-related data, four age groups were distinguished: 30–45, 46–60, 61–75, and over 75 years.

Any potentially negative aspects of photodynamic treatment from the patient's point of view were also investigated. The patients were asked to assess the character of pain and burning or pricking sensations associated with PDT using a 0–3 scale.

The collected data were statistically analyzed with the use of programs: Statistica for Windows (StatSoft, USA) and Excel (Microsoft, USA). To assess differences in the lesion size between groups, the Wilcoxon signed–rank test was used, and to determine differences in the lesion size before and after treatment, the paired Student's *t* test. The differences were considered statistically significant at *p* ≤ 0.05.

## Results

On baseline, the mean size of the OPL lesion was 6 cm^2^ ± 4.5. The lesions localized on the cheeks and lips were larger (6.6 cm^2^ ± 4.63) as compared to those observed on the gums and tongue (3 cm^2^ ± 1.9). The lesions with sizes classified into group 3 and group 1 were the most common. In smokers the OLP lesions were approximately twice as large as in nonsmokers. Those on the cheeks and lips were similar in women and men, whereas on the gums and tongue, they were approximately twofold larger in men.

**Fig. 1:**
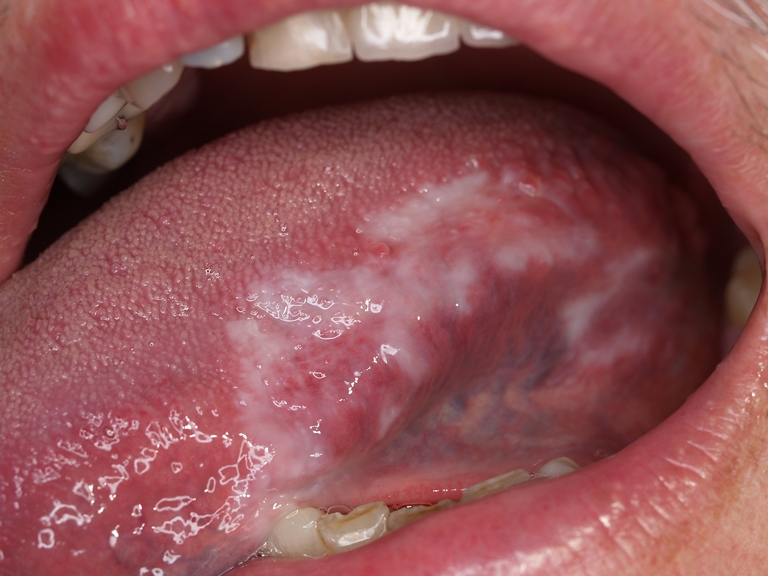
OLP of the tongue. A 38-year-old male, smoker, status before PDT

**Fig. 2:**
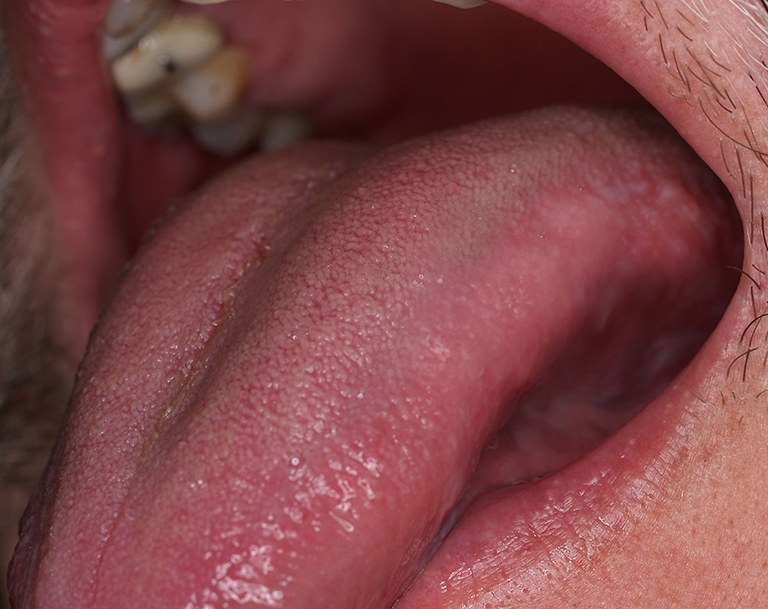
Patient from Fig. 1 after PDT—complete regression

After treatment, improvement was observed in 39 sites, including 14 with complete regression—13 on the cheeks and lips and 1 on the gums and tongue (Figs. 1 and 2). In nine sites, improvement was not evident—in five on the cheeks and lips and in four on the gums and tongue. The mean reduction in the size of lesions in OLP patients was statistically significant (55 %) (*p* = 0.00007366). Also statistically significant was the reduction in the lesions on the cheeks and lips (57.6 %) both in men and women, and smokers and nonsmokers (on the borderline of significance). The OLP lesions on the gums and tongue were reduced after treatment, the mean reduction being 30 %, but the differences were not statistically significant (*p* = 0.56050000). Detailed data have been presented in Tables 1 and 2.

**Table 1:** Mean size, standard deviation, and mean reduction in OLP lesion size before and after treatment

Site of lesion	Number of lesions	Mean size of lesions (cm^2^)				Mean reduction in lesions (%)	*p*—borderline significance
		Before treatment	SD	After treatment	SD		
Lichen planus	48	6	4.5	2.7	2.62	55.0	0.00007366
Cheeks and lips	40	6.6	4.63	2.8	2.81	57.6	0.00006788
Women	28	6.3	4.27	2.8	2.78	55.6	0.00069380
Men	12	7.4	5.29	2.9	2.87	60.8	0.01793000
Smokers	4	11.4	5.44	4.8	0.82	57.9	0.05714000
Nonsmokers	36	6.1	4.21	2.6	2.87	57.4	0.00015430
Gums and tongue	8	3	1.90	2.1	1.21	30.0	0.56050000
Women	7	2.6	1.63	1.9	1.05	26.9	0.66550000
Men	1	6	0	4	0	33.3	–
Smokers	2	4.5	1.50	2.5	0.5	44.4	0.66670000
Nonsmokers	6	2.5	1.75	2	1.35	20.0	0.72730000

**Table 2:** Number and percentage of OLP lesions before and after treatment

Site of lesion	Number of lesions						
	All	Cured		Partly cured		Unchanged	
Lichen planus	48	14	29.17 %	25	52.08 %	9	18.75 %
Cheeks and lips	40	13	32.50 %	22	55.00 %	5	12.50 %
Women	28	8	28.57 %	15	53.57 %	5	17.86 %
Men	12	5	41.67 %	7	58.33 %	0	0.00 %
Smokers	4	0	0.00 %	4	100.00 %	0	0.00 %
Nonsmokers	36	13	36.11 %	18	50.00 %	5	13.89 %
Gums and tongue	8	1	12.50 %	3	37.50 %	4	50.00 %
Women	7	1	14.29 %	2	28.57 %	4	57.14 %
Men	1	0	0.00 %	1	100.00 %	0	0.00 %
Smokers	2	0	0.00 %	2	100.00 %	0	0.00 %
Nonsmokers	6	1	16.67 %	1	16.67 %	4	66.67 %

Following treatment, the mean lesion reduction within the cheeks and lips was greatest in group 1 (63.6 %) and smallest in group 3 (50.8 %). The mean reduction within the gums and tongue was greatest in group 3 (41.7 %) and smallest in group 2 (16.7 %). As shown by the analysis of the lesions in the respective size groups, the mean reduction in OLP lesions on the cheeks and lips was greater as compared to the gums and tongue (Table 3).

**Table 3:** Surface area and mean reduction in OLP lesions before and after treatment in the respective size groups

Size measurement	Location					
	Gums and tongue			Cheeks and lips		
	Before (cm²)	After (cm²)	Mean size reduction	Before (cm²)	After (cm²)	Mean size reduction (%)
Group 5 (*n* = 2)	–	–	–	20	8 ± 4	55.0
Group 4 (*n* = 7)	–	–	–	12	4 ± 4	63.1
Group 3 (*n* = 16)	6	3 ± 1	41.7 %	7.5 ± 1.5	3 ± 3	50.8
Group 2 (*n* = 8)	3 ± 0.5	3 ± 1	16.7 %	4 ± 1	2 ± 1.5	57.8
Group 1 (*n* = 15)	1.5 ± 0.5	1.5 ± 1.5	25.0 %	1.5 ± 1	1 ± 1	63.6

Interestingly, after treatment, no lesions were found to be extensive (>15 cm^2^), and only one lesion was 10–15 cm^2^ in size. All the other lesions were classified into group 2 and group 3 (3–10 cm^2^) (Table 4).

**Table 4:** Number of OLP lesions before and after treatment in the respective size groups

Measurement before treatment	Measurement after treatment					
	Group 5	Group 4	Group 3	Group 2	Group 1	Group 0
Group 5 (*n* = 2)	–	1	1	–	–	–
Group 4 (*n* = 7)	–	–	3	2	–	2
Group 3 (*n* = 16)	–	–	2	10	2	2
Group 2 (*n* = 8)	–	–	–	2	4	2
Group 1 (*n* = 15)	–	–	–	–	7	8

*n* number of lesions in the respective size range

With respect to gender, PDT efficacy for the lesions on the gums and tongue was similar in women and men (30.6 and 33.3 %, respectively). However, for the lesions on the cheeks and lips, PDT had a more beneficial effect in men than in women (51.4 and 68.9 %, respectively).

With respect to the patients' age, the treatment of lesions on the cheeks and lips was most effective in patients over 75 (66.9 %), whereas least effective in patients aged 61–75 years (50.9 %). As OLP lesions were not found on the gums and tongue in all age groups, it was impossible to make a complete statistical analysis. They were only observed in the group of patients aged 46–60, and their treatment efficacy was 33.3 % (Table 5).

**Table 5:** Comparison of the efficacy of treatment for OLP in the respective age groups with regard to the lesion size

Size measurement	Location and age group							
	Gums and tongue				Cheeks and lips			
	30–45 years	46–60 years	61–75 years	Over 75 years	30–45 years	46–60 years	61–75 years	Over 75 years
Group 5 (*n* = 2)	–	–	–	–	70.0 %	–	–	70.0 %
Group 4 (*n* = 7)	–	–	–	–	66.7 %	47.2 %	–	77.8 %
Group 3 (*n* = 16)	–	41.7 %	–	–	25.0 %	58.0 %	31.7 %	52.8 %
Group 2 (*n* = 8)	–	33.3 %	0.0 %	–	16.7 %	–	71.1 %	–
Group 1 (*n* = 15)	0.0 %	50.0 %	–	0.0 %	100.0 %	57.1 %	50.0 %	–
Total (*n* = 48)	0.0 %	33.3 %	0.0 %	0.0 %	55.7 %	54.1 %	50.9 %	66.9 %

As shown by questionnaire survey, none of the patients complained of any discomfort during the PDT sessions. They all located pain sensations at the level of 0 according to the subjective scale.

## Discussion

The analysis of literature data clearly indicates that there is no standard protocol of OLP lesion treatment which would be predictable and effective. That is why there are still searches done for new methods of treatment that would be more effective and possibly noninvasive, e.g., photodynamic therapy. The first photosensitizers used in PDT were compounds belonging to the group of hematoporfirin, photofrin, and meta-tetra (hydroxyphenyl) chlorin. Later, for a relatively long time, the photosensitizers of choice included 5-aminolevulinic acid (ALA) and dyes—toluidine blue and methylene blue [16, 17]. So far, there is no photosensitizer which would meet all clinical demands. Majority of them have disadvantages like: prolonged skin sensitivity to light, limited cell selectivity, and unpredictable efficacy [18]. In our study Photolon^®^ was chosen as its properties seem to be advantageous as compared to other commonly used photosensitizers. Its absorption of light with increased wavelength leads to better therapeutic effect, because the photosensitizer can reach deeper localized lesions without increase of phototoxicity [19–21]. Research performed by Mohamed Ali-Seyed et al. [22] in 2011 proved that Photolon preferentially localizes in intracellular organelles in the following sequence: nucleus, mitochondria, lysosomes, and Golgi apparatus. Elimination of cancerous cells is done by induction of CT-26 cell apoptosis which has physiological meaning and may be related to low drug toxicity. Anticancerous properties of Photolon were proved in studies of other authors [10, 21]. However, we have not found in the available literature any reports concerning the use of this photosensitizer for the treatment of oral lichen planus, which makes any comparison impossible.

Studies concerning the use of other photosensitizers in the treatment of OLP are also rare. Aghahosseini et al. [17] assessed PDT as an alternative method for the treatment of lichen planus in 13 patients with 26 mucosa lesions. The patients rinsed their mouths for 5 min with 5 % aqueous solution of methylene blue. After 10 min the lesions were illuminated using a low-energy laser of 632 nm wavelength and exposure dose of 120 J/cm^2^. Improvement was noted in 16 lesions and complete remission in 4. The mean reduction in the size of lesions was 44.3 %. In another publication the same authors described the application of PDT in two patients with five lichen planus lesions resistant to previous treatment. The procedure using methylene blue as a photosensitizer caused improvement in four cases—in two cases the lesions disappeared, and in the other two they were reduced by 50 % [23].

In the present study we obtained lesion remission or reduction in approximately 81 % of OLP cases. There was no response to treatment in 18.75 % of patients. PDT was more effective in cases of lesions developing on the cheeks and lips as compared to the tongue and gums. The percentage of lesions on the cheeks and lips that remained unchanged after PDT was 12.5 %, whereas on the tongue and gums, it was as high as 50 %. However, no definite conclusions can be drawn from the data due to a very small number of lesions located on the gums and tongue.

The size of clinical lesions in patients undergoing PDT decreased significantly in the case of OLP (55 %). The treatment had the best effects in patients with OLP situated on the cheeks and lips, where mean size reduction was 57.6 %. A smaller reduction was observed within the masticatory mucosa, i.e., on the tongue and gums (30 %). This observation is difficult to interpret and needs to be confirmed with a larger number of cases.

Nevertheless, the percentage of cured or partially cured lesions (81 %) in our study was similar to Aghahosseini et al.'s [17] (76.9 %), and the protocol used in the presented study seems to be superior. Patients included in our study had severe lesions even over 15 cm^2^ (average, 6 cm^2^ ± 4.5). In the study of cited authors, lesions were small (1.8 cm^2^ ± 0.7) which in our study would be classified as group 1—the most mild lesions. After treatment average lesion size was 2.7 cm^2^ ± 2.62 which gave an area reduction of 3.3 cm^2^. In Aghahosseini et al.'s study, after treatment, mean lesion size was 1 cm^2^ ± 0.9 which gave an area reduction only of 0.8 cm^2^. Moreover, the method of measurement in this study was questionable. It was done with the use of a tongue blade on which a handmade scale with a different range was drawn. Repeatability of measurements was not accurate as may be clearly seen in photos presented in this article. Also, even side effects were not a significant problem; however, few patients complained about a mild burning sensation.

The relevant finding of our study was that the efficacy of PTD was comparable in women and men irrespective of age. The analysis of age-related PDT efficacy revealed that it was slightly higher in patients over 46 years of age than in younger subjects. Besides, since PDT procedures do not cause any unpleasant sensations and thus do not require administration of analgesics or anesthetics, they can be particularly recommended for the elderly. As the therapy has no side effects, the session can be repeated whenever recurrence takes place. This is especially important in OLP which is a chronic disease with often no predictable recurrences. The lack of side effects as compared to other PDT protocols, especially with the use of ALA which is painful, can be beneficial particularly in patients with long-term course of disease [18].

## Conclusions

The mean 55 % reduction of lesions obtained in the study favors the introduction of PDT as an alternative therapy for OLP. However, due to a limited number of relevant literature data, the method requires further verification with respect to its efficacy and possibility of long-term remission of the disease.
